# Methodological Considerations in Analyzing Anthropometric Measures in Japan

**DOI:** 10.2188/jea.JE20250690

**Published:** 2026-11-05

**Authors:** Fumi Oono, Anna Kinugawa, Sachiko Ono

**Affiliations:** 1 Department of Eat-loss Medicine, Graduate School of Medicine, The University of Tokyo, Tokyo, Japan; 2 Department of International and Community Oral Health, Tohoku University Graduate School of Dentistry, Sendai, Japan

Anthropometric measures are widely used in epidemiologic research, and their ease of measurement facilitates their application in public health practice. Nevertheless, the associations between these measures and health outcomes may be non-linear and non-monotonic, including J-shaped associations, which warrant careful analytical handling. In this letter, we use a recent study as an example to highlight the importance of considering such non-linear and non-monotonic associations. Although these methodological considerations are broadly applicable across populations and over time, they are particularly relevant in Japan, where the distributions of anthropometric measures are generally lower, and a larger proportion of individuals are in the lower range of anthropometric measures compared with Western populations. For example, according to the World Health Organization, the prevalence of underweight among adults in 2022, defined as a body mass index (BMI) <18.5 kg/m^2^, was 2.0–2.1% in the United States and the United Kingdom, compared with 10.8% in Japan.^1^ Underweight is especially prevalent among young Japanese women, with more than 20% of women aged 20–29 years having a BMI <18.5 kg/m^2^.^2^ These population-specific characteristics may not be fully appreciated by international reviewers and readers. Therefore, Japanese researchers should conduct and report analyses of anthropometric measures with particular caution.

Recently, a study published in the *American Journal of Clinical Nutrition*, one of the leading journals in nutrition research, compared traditional and novel anthropometric indices with respect to their ability to predict cardiovascular disease (CVD) incidence among Japanese adults.^3^ All indices, however, were modeled as linear predictors across the main and sensitivity analyses without evaluating potential non-linearity. Under this linear assumption, differences in estimates (eg, hazard ratios and areas under the curve) may merely reflect how well each index fits a presumed linear association rather than true differences in predictive performance. Given that some studies suggest that underweight is also associated with higher CVD incidence^4^^,^^5^ and mortality,^6^ the underlying associations may be non-linear and non-monotonic. Thus, individuals with the lowest anthropometric indices do not necessarily have the lowest CVD risk, and linear modeling may obscure elevated risk at lower levels of anthropometric indices. Moreover, when the true exposure–risk association is J-shaped, fitting a single linear model leads to systematic misestimation across the exposure range. As illustrated in Figure 1, a linear model underestimates risk at both the low and high ends of the exposure distribution, while overestimating risk around the nadir, where the true risk is lowest. This pattern reflects the averaging imposed by linearity, which cannot accommodate opposing gradients in different regions of the exposure range. Such misestimation is expected to be more pronounced in populations where low anthropometric measures are relatively common. Examining non-linear and non-monotonic associations would therefore be more appropriate, for example, through the use of spline-based methods or categorizations.^7^

**Figure 1.**
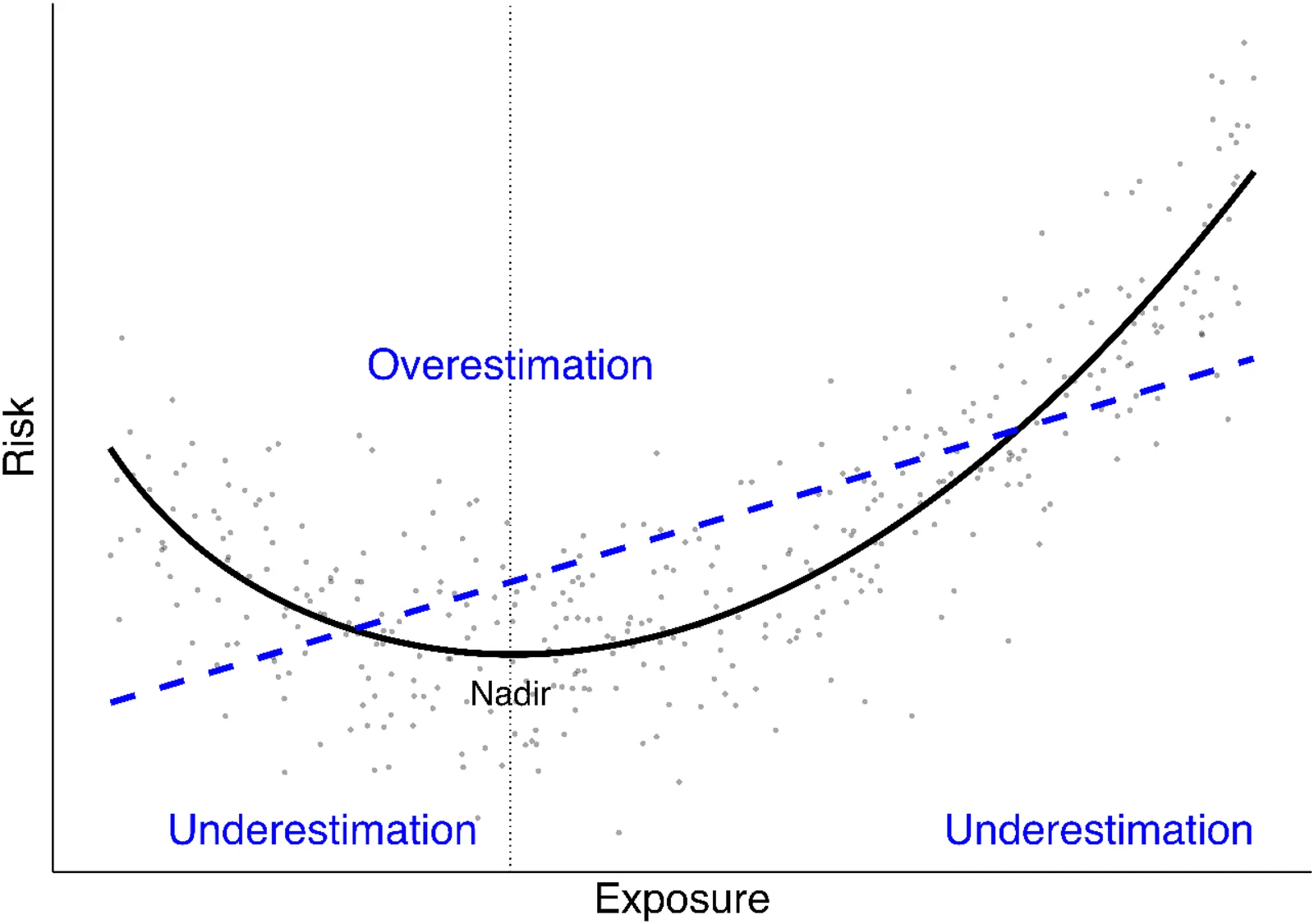
Systematic misestimation induced by a linear model under a J-shaped association. Data are simulated for illustration (*n* = 400). Grey points represent simulated observations; the solid black curve denotes the prespecified true J-shaped exposure-risk association; the dashed blue line denotes the fitted linear regression.

Nevertheless, categorization of anthropometric variables also warrants careful consideration, even when standardized cut-off points are used. Categorization implicitly assumes negligible variation in outcomes within each category.^8^^,^^9^ As a familiar example, categorizing BMI simply at <18.5 kg/m^2^ treats individuals with BMI values of 18.4 kg/m^2^ and 15.0 kg/m^2^ as equivalent, despite substantial differences in clinical severity.^10^ For example, a meta-analysis reported that the relative risks of all-cause mortality (reference BMI: 23 kg/m^2^) were 1.47 at a BMI of 17.5 kg/m^2^, 1.83 at 16.0 kg/m^2^, and 2.24 at 15.0 kg/m^2^.^11^ Although simple cut-off points are often necessary in public-health practice for screening and policy development, research on anthropometric measures should not rely solely on rough categorization. Researchers can examine associations using flexible continuous modeling, such as spline models. Large-scale studies, including cohort consortia and real-world datasets with health check-up records, offer valuable opportunities to examine how risk varies across the lower end of the anthropometric range. In this context, Japanese data are particularly informative, given the relatively high prevalence of underweight.

In conclusion, relying solely on linear models or rough categorization is insufficient when analyzing the associations between anthropometric measures and health outcomes, especially in Japan. The distinctive distribution of anthropometric measures in Japan provides an opportunity to examine risk at the lower end of the anthropometric range and to address the question of how low is too low. Evidence generated from such analyses can inform public-health practice in Japan and globally.
